# Evaluating context-specific evidence-based quality improvement intervention on lymphatic filariasis mass drug administration in Northern Ghana using the RE-AIM framework

**DOI:** 10.1186/s41182-021-00305-3

**Published:** 2021-02-18

**Authors:** Alfred Kwesi Manyeh, Tobias Chirwa, Rohit Ramaswamy, Frank Baiden, Latifat Ibisomi

**Affiliations:** 1 Division of Epidemiology and Biostatistics, School of Public Health, University of the Witwatersrand, Parktown, Johannesburg, South Africa; 2 Institute of Health Research, University of Health and Allied Sciences, Ho, Volta Region, Ghana; 3 Public Health Leadership Program, Gillings School of Global Public Health, University of North Carolina, 4107, McGavran-Greenberg Hall, Chapel Hill, NC USA; 4 Faculty of Infectious and Tropical Diseases, London School of Hygiene and Tropical Medicine, London, UK; 5 Nigerian Institute of Medical Research, Yaba, Lagos State Nigeria

**Keywords:** Lymphatic filariasis, Quality improvement, Mass drug administration, RE-AIM, Northern Ghana

## Abstract

**Background:**

Over a decade of implementing a global strategy to eliminate lymphatic filariasis in Ghana through mass drug administration, the disease is still being transmitted in 11 districts out of an initial 98 endemic districts identified in 2000. A context-specific evidence-based quality improvement intervention was implemented in the Bole District of Northern Ghana after an initial needs assessment to improve the lymphatic filariasis mass drug administration towards eliminating the disease. Therefore, this study aimed to evaluate the process and impact of the lymphatic filariasis context-specific evidence-based quality improvement intervention in the Bole District of Northern Ghana.

**Method:**

A cross-sectional mixed methods study using the RE-AIM (Reach, Effectiveness, Adoption, Implementation, and Maintenance) framework to evaluate the context-specific evidence-based quality improvement intervention was employed. Quantitative secondary data was extracted from the neglected tropical diseases database. A community survey was conducted with 446 randomly selected participants. Qualitative data were collected from 42 purposively selected health workers, chiefs/opinion leaders and community drug distributors in the study area.

**Results:**

The evaluation findings showed an improvement in social mobilisation and sensitisation, knowledge about lymphatic filariasis and mass drug administration process, willingness to ingest the medication and adherence to the direct observation treatment strategy. We observed an increase in coverage ranging from 0.1 to 12.3% after implementing the intervention at the sub-district level and reducing self-reported adverse drug reaction. The level of reach, effectiveness and adoption at the district, sub-district and individual participants’ level suggest that the context-specific evidence-based quality improvement intervention is feasible to implement in lymphatic filariasis hotspot districts based on initial context-specific needs assessment.

**Conclusion:**

The study provided the groundwork for future application of the RE-AIM framework to evaluate the implementation of context-specific evidence-based quality improvement intervention to improve lymphatic filariasis mass drug administration towards eliminating the disease as a public health problem.

## Background

### Lymphatic filariasis mass drug administration

Lymphatic filariasis (LF) commonly known as elephantiasis is a mosquito-borne incapacitating and disfiguring disease caused by three species of parasitic worms (*Wuchereria bancrofti*, *Brugia malayi* and *B. timori*). LF is rarely fatal, but it causes lymphedema, elephantiasis and hydrocele at the clinical stage. These conditions cause severe pain and result in permanent disability, social exclusion and loss of productivity [1]. LF has been endemic in Africa, Asia, the Pacific and the Americas historically [2]. Approximately one billion people from 72 countries were at risk for LF infection, with nearly 36 million people being affected by LF-associated morbidity before the World Health Organization (WHO) established the Global Program to Eliminate Lymphatic Filariasis (GPELF) in 2000 [3]. The goal of the GPELF is to eliminate LF in all disease-endemic areas by 2020.

The main approach recommended by GPELF was based on reduction of microfilaria (mf) prevalence in endemic communities below < 1% through mass drug administration (MDA) of anti-filarial medicine. The recommended anti-filarial medicine for sub-Saharan Africa is either diethylcarbamazine or ivermectin and albendazole. This strategy is recommended to be implemented for at least 5 years with effective population treatment coverage of ≥ 65%. This is because the life expectancy of an adult worm is from 4 to 6 years. It is therefore expected that the disease transmission should be interrupted after 5 years of effective treatment coverage.

The main GPELF strategy to interrupt transmission is MDA using combinations of two anti-filarial medicines (albendazole plus either diethylcarbamazine or ivermectin) delivered once yearly to entire eligible populations in endemic areas. The recommended regimen in sub-Saharan Africa is either diethylcarbamazine or ivermectin and albendazole for a minimum of 5 years with effective population treatment coverage (≥65%).

Ghana became one of the first West African countries to implement MDA intervention through the Ghana Filariasis Elimination Program (GFEP). The implementation of this evidence-based intervention started in 10 endemic districts in 2001 and reached national coverage by 2006.

The GFEP aims to reduce LF prevalence level to below 1% after 4–6 rounds of high-coverage (>80%) MDA [4, 5].

Over a decade of implementing the MDA intervention in Ghana, the disease transmission persists in 11 districts out of an initial 98 endemic districts. These 11 districts have microfilariae prevalence rate above the 1% threshold, there is a need for interruption of the disease transmission and these are now termed LF “hotspot” districts [6]. Bole District in Northern Ghana is one of the districts with the highest microfilariae prevalence in Ghana [5]. In 2016, microfilariae prevalence in Bole District was 1.9% with some communities having a prevalence rate as high as 5.9% [5].

### The context-specific evidence-based quality improvement intervention

**Fig. 1:**
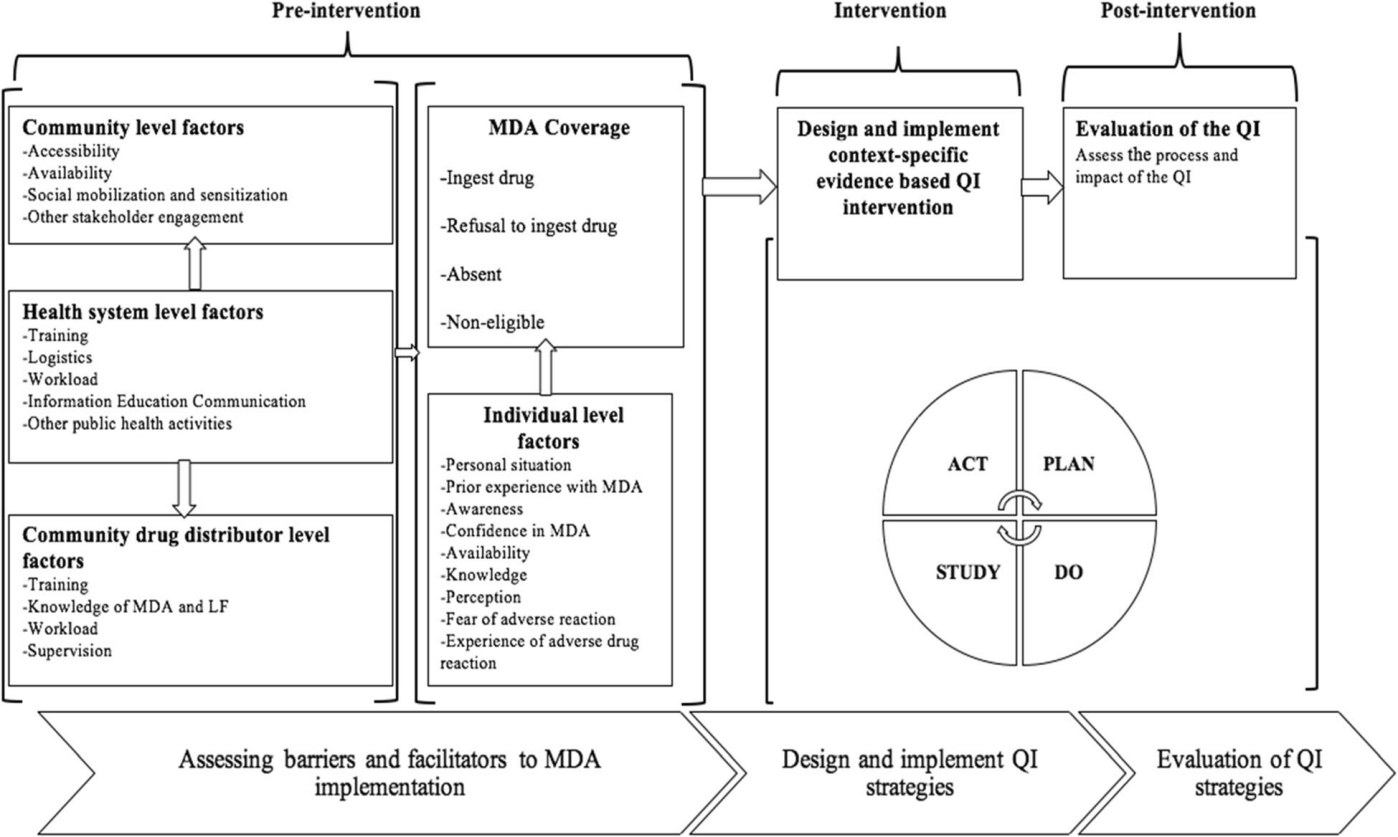
Conceptual framework showing the connection between the study phases

In 2017, TDR, the Special Program for Research and Training in Tropical Diseases, funded a project to conduct implementation research (IR) towards eliminating LF in the Bole District of Northern Ghana. An initial needs assessment to identify implementation bottlenecks associated with the LF MDA intervention in the Bole District of Ghana was conducted using a mixed methods approach. This strategy was used to gain a deeper understanding of the critical issues affecting the implementation of the LF MDA from the perspective of the services providers (health workers) and frontline workers (community drug distributors), stakeholders (community leaders) and the clients (community members) [5]. The framework connecting all three (3) phases of the study is detailed in Fig. 1.

The results of the pre-intervention phase indicated that the continuous transmission of LF in the Bole District is characterised by refusal to take the drug due to poor knowledge and misconceptions of the disease, poor community mobilisation and sensitisation, the fear of adverse drug reactions, non-adherence to the directly observed treatment strategy, poor adherence to the MDA protocol and non-participants’ responsiveness as detailed elsewhere [5].

Based on the findings of the pre-intervention phase, a context-specific evidence-based quality improvement (CEQI) intervention was designed and implemented using Intervention Mapping (IM) and Plan-Do-Study-Act (PDSA) approaches [5]. The CEQI intervention was based on four (4) key strategies namely training of community drug distributors (CDDs), social mobilisation and sensitisation, the involvement of community leaders and other stakeholders and improving the drug distribution process. These strategies were described in seven (7) domains: actor, the action, action targets, temporality, dose, implementation outcomes addressed and theoretical justification, as shown in Table 1 [5].

**Table 1:** Description of components of the context-specific evidence-based quality improvement intervention

Domain	Strategy: training of community drug distributors (CDDs)	Strategy: social mobilisation and sensitisation	Strategy: involvement of community leaders and other stakeholders	Strategy: drug distribution process
**Actor(s)**	• District director, sub-district NTD focal persons, disease control officers and implementation/QI team (district and sub-district NTD focal persons and heads, DCO, HPO, Assemblymen/women and PI) who have been trained.	• Intervention implementation/QI team (district and sub-district NTD focal persons, opinion, religious and traditional leaders and PI).	• Intervention implementation/QI team	• Intervention implementation/QI team and CDDs
**Action(s)**	• Train drug distributors to have a good understanding of the programme and to be able to instil the same knowledge to the community members. • Train drug distributors to be able to convince every qualified person in the endemic area to participate in the MDA exercise. • Train drug distributors on the inclusion and exclusion criteria of the MDA. • Train drug distributors on the possible adverse drug reactions and be able to explain them to the community members. • Instilling the skills of communication and interaction to the drug distributors and the importance of being patient and tolerant with difficult community members. • Vigorous enforcement of the MDA procedures, in particular, DOT policy during training and supervision.	• An evidence-based, multi-channel communication strategy to result in high levels of awareness among community members, (radio discussions and announcements, announcements in churches, mosques, schools, etc., community durbars and meetings with social groups to explain MDA relevance and public/community announcements). • Focus key messages on cause and mode of transmission of the disease, importance of the MDA and how to identify, what to do and minimise adverse drug reactions.	• Community/opinion leaders such as Chiefs, Assemblymen/women, religious and traditional leaders should be involved in the MDA exercise.	• An adequate number of days should be dedicated to the distribution exercise (not less than 1 week) • The distribution should reach people in institutions, markets, places, offices and homes. • People with higher-level qualifications and a good knowledge of the MDA should be sent to institutions and offices to distribute the drug. • Strong enforcement of the DOT policy.
**Target (s) of the action**	• Drug distributors in the endemic communities.	• People in the endemic communities.	• Community leaders.	• People in the endemic communities.
**Temporality**	• The drug distribution should start within 1 week after the training of drug distributors.	• Social mobilisation and sensitisation should start 2 weeks before and should continue during the MDA.	• Before, during and after MDA exercise.	• During the drug distribution
**Dose**	• The training of the CCDs should be detailed enough to equip them well for the MDA task, and the training period should not be more than 1 day to enhance their active participation in the training.	• Each endemic community should have at least two social mobilisation and sensitisation exercises (community durbar, school education, information centre announcement, education at church and mosque, and or radio talk shows etc.) for the start of MDA and at least one during MDA.	• Every endemic community must have a community leader representing it.	• The distribution exercise should not be less than 1 week in the endemic district. • Over 80% of the people in the endemic communities must be covered.
**Implementation outcome(s) and effect**	• Increase the level of adherence to LF MDA implementation procedures and participants’ responsiveness.	• At least 15% increase in MDA coverage and reduction in the number of refusals (increased participant responsiveness).	• At least 15% increase in MDA coverage and reduction in the number of refusals (increased participant responsiveness).	• At least 15% increase in MDA coverage and reduction in the number of refusals (increased participant responsiveness).
**Justification**	• Researchers suggest that drug distributors are the interface between MDA programs and their targeted population; hence, their adequate training is crucial to the success of the MDA [7–10].	• It has been shown that evidence-based, context-specific and multi-channel social mobilisation and sensitisation is required for the LF elimination programme to succeed [4, 10, 11].	• Stakeholder engagement and involvement in LF MDA cannot be overemphasised [4, 9, 10].	• The MDA implementation process is crucial for participants’ responsiveness to the program [4, 10].

*CDD* community drug distributors, *DCO* disease control officer, *HPO* health promotion officer, *QI* quality improvement, *DOT* direct observed treatment, *MDA* mass drug administration [5]

As part of the monitoring process and to ensure successful implementation and sustainability of the CEQI, a CEQI implementation team with representation from the district and the sub-districts was formed. The implementation team’s essential obligation was to ensure the smooth implementation and sustainability of the CEQI intervention. Besides, a WhatsApp group was created to link members of the implementation team. The group platform facilitates sharing ideas and resolving challenges faced during the CEQI intervention implementation, as shown elsewhere [5]. The WhatsApp platform was also used by the principal investigator (the first author) to review work progress and reinforce compliance with the components of the CEQI.

Reach, Effectiveness, Adoption, Implementation, Maintenance (RE-AIM) framework was used to guide the evaluation of the CEQI intervention implementation. The RE-AIM framework was developed to assess the effects of public health interventions [12].

*Reach* is defined as the proportion of eligible individuals in the aimed population who partook in an intervention program and the extent to which those individuals represent the target population. *Effectiveness* is the degree to which the intervention program positively affects an outcome(s) of interest. *Adoption* is a measure of the total number of program providers who implement an intervention and the extent to which they represent all potential program providers. *Implementation* is an organisational measure of the quality of the intervention delivery and its adherence to the research program’s essential elements. *Implementation* is sometimes referred to as *treatment fidelity*. *Maintenance* is the measure of the program’s effectiveness in attaining the expected outcome for an extended time. It is also a measure of the intervention’s sustainability and indicates whether the intervention is likely to become an institutional culture [12, 13].

The RE-AIM has been useful in evaluating intervention in diverse fields including behavioural change interventions [14, 15], weight loss studies [16, 17], nutrition projects [18], studies on injury prevention [19, 20] and studies on manual activity [18, 21]. The RE-AIM framework has been applied to assess a single intervention’s effectiveness at employee, community and patient levels [22]. The framework has also been useful in studying the impact of evidence-based interventions with fidelity and interventions that focus on improving organisational culture and adherence to clinical guideline [23]. It has also been used to assess the impact of a single intervention within a context of a broader project [24] and recently to evaluate more extensive multidimensional interventions [13].

Qualitative, quantitative and mixed methods approaches to applying the RE-AIM framework have been demonstrated in evaluating interventions [25]. With awareness of these various options of applying the RE-AIM framework, the primary purpose of this paper is to use the RE-AIM framework to evaluate CEQI intervention’s effect on the implementation of LF MDA in the Bole District of Northern Ghana.

## Methods

### Study design and data source

We used mixed methods study design, which involves extracting quantitative secondary MDA data from the neglected tropical diseases database in Bole District Health Administration in the Northern Region of Ghana, a community survey and qualitative data collection. The post-CEQI survey data were collected from randomly selected community members.

### Sample size and sampling procedure

The minimum sample size (*n*) for the primary quantitative data was determined using the formula: *n* = (*Z*_α/2_)^2^ × (*p* × *q*)**/***d*^2^ adopted from World Health Organization’s practical manual for sample size determination in health studies [26]. This formula was also used in a similar LF compliance study conducted in Ghana [27]. The sample size calculation was based on the assumption that 65% [27] of the population was aware of the MDA program and ingested the drugs, *Z*_α/2_ is the confidence level at 95% = 1.96, *p* is the coverage, *q* is (1-*p*) and *d* is the level of precision. Here *p* = 65% and *d* = 5%. The sample size was increased by 30% to compensate for nonresponse and missing information.

Therefore,

$$ \mathrm{n}=\frac{(1.96)^2\ast \left(0.65\ast 0.35\right)/{(0.05)}^2}{=349.58\ \mathrm{People}.} $$

$$ \frac{\mathrm{A}\ \mathrm{further}\ 30\%=349.58\ast 0.3}{=104.88} $$

Hence,

$$ \frac{\mathrm{the}\ \mathrm{actual}\ \mathrm{sample}\ \mathrm{size}=349.58+104.88}{=454\ \mathrm{people}.} $$

However, five and three participants in the pre and post-intervention respectively do not have complete information; hence, 222 and 224 participants were respectively included in the pre- and post-intervention analysis. The selection process of the survey participants is described elsewhere [5].

The survey data includes information on socio-demographic characteristics, knowledge of how LF is acquired, knowledge of signs associated with LF, knowledge of LF prevention and misconceptions about LF [28].

These variables were measured by correct answers to signs associated with LF, LF prevention methods and how LF is acquired. The method for measuring these variables and estimating comprehensive knowledge of LF is detailed elsewhere [28].

The quantitative data include information on pre- and post-CEQI intervention MDA coverage and self-reported adverse drug reaction. We also extracted information from weekly written reports submitted by sub-district heads of health services during the implementation of the CEQI.

The qualitative data was collected from purposively selected participants through in-depth interviews (IDIs) using semi-structured interview guides. The interview guides were pre-tested in a different rural LF endemic district with similar settings as the study district. The IDIs were conducted with 42 participants, including community leaders, community drug distributors (CDD) and health workers. The health workers include district and sub-district directors of health services, disease control officers, health information officers and nurses. The qualitative arm participants were purposively selected from six (6) sub-districts to gather enough information to evaluate the effect of the intervention. Except for health worker IDIs, all data collection tools were translated into the predominantly spoken local language in the study area. Different individuals in the study area were selected to participate in the pre- and post-intervention primary data collection.

The data was collected between March and April 2018 by trained graduate research assistants.

All interviews were recorded using digital voice recorders with the consent of the study participants. Detailed field notes and training registers were turned into data documents for analysis.

The interviews with health workers were conducted in English while those of CDDs and community leaders were held in the local languages and translated into English by two independent language experts during transcription.

### Measurements

#### Reach

Reach was assessed at the district, sub-district, CDD and the individual community member levels.

*District level*: Reach was measured as the proportion of the district management team members who participated in the training before the 2017 drug distribution.

*Sub-district level*: Proportion of sub-district management team who participated in the training of the CDDs’ training before 2017 MDA.

*Individual community member level*: This was assessed using the number of people who received the drug (coverage) during the 2017 MDA.

#### Effectiveness

Multiple data sources through the mixed methods research approach were used to measure the effectiveness of the intervention on the level of knowledge about the LF, understanding of the MDA among the study participants, the involvement of community leaders and the MDA coverage.

#### Adoption

We assessed the level of adoption as the proportion of sub-districts that implemented each CEQI intervention component. Through interviews with the study participants, we assessed their knowledge level to ascertain understanding and information given by the interviewers (CDDs, health workers) on the program. If the interviewers determined that the program was not implemented correctly, they were asked the impediments during the implementation and how they planned to overcome them.

#### Implementation

The level of implementation was assessed (i.e. fidelity to delivering the CEQI intervention) with the study participants. This was done through IDIs to explore the level of information they have been given and determine how well sub-districts adhered to strategies during the implementation. We also assessed the activities carried out during the implementation of the CEQI from the weekly report submitted by the sub-districts heads of health services and from the WhatsApp platform.

#### Maintenance

The assessment of the district-, sub-district- and CDD-level maintenance of the intervention was used to determine the extent to which the CEQI intervention can become integrated into the LF MDA’s routine activities.

We also determined the extent to which the intervention implementation team could continue and sustain the intervention activities. A review of barriers and possible solutions to enable the successful implementation of the intervention from the perspective of the participants (through the IDIs and from weekly written reports) was carried out.

## Results

### Reach

Before the 2017 MDA commencement, respondents reported that training, which usually lasts between 1 and 3 days, was held for CDDs. The training focused on teaching CDDs how to administer and handle the MDA drugs and filling the forms. They were also trained on community members’ sensitisation and convinced individuals who were resistant to taking the pills. Respondents also indicated they were taught to identify MDA illegible community members and ensure all houses are visited during the exercise. The participants reported that training was practical; they were provided with material (books and pictures) of the disease to educate community members, which helped facilitate their work.

We were trained for three days, and the training was very good. We were taught a lot about LF and drug distribution. We were also taught how to sensitise our community members to participate in the drug administration (CDD, Tinga).

#### District level

The district director of health service, district disease control officer, district public health officer and district public health nurse participated in the training sessions. This participation represents 100% reach for the district-level officials who superintends the MDAs in the district.

#### Sub-district level

The heads of each of the five (5) sub-districts and their disease control officers participated in the training. Thus, 10 (100%) representation of the sub-districts officials who oversee the implementation of the MDA at the sub-district level. Five (5) nurses from the sub-districts also participated in the training to supervise the CDDs during the MDA.

#### Community drug distributor level

A total of 152 CDDs were trained representing 98% of the total CDDs identified for the MDA in the study district. Three (3) were absent due to ill health, but the arrangement was made for them to be trained at the sub-district level before the commencement of the 2017 MDA.

#### Individual community member level

A total of 66623 (83.4%) eligible community members received the LF medication in the study district during the 2017 MDA.

#### Community leader level

Two (2) key community leaders from each sub-district (a total of 10) participated in the CDD’s training. The community leaders were also involved in the social mobilisation and sensitisation activities in their various communities in the sub-districts.

Although there is a reduction of 3.2% in MDA coverage in Bole sub-district after the intervention shown in Table 2, all other sub-districts recoded an increase in coverage ranging from 0.1 to 12.3% after the implementation of the intervention.

**Table 2:** Distribution of pre- and post-intervention sub-district MDA coverage among residents in Bole District

Sub-districts^a^	Pre-intervention % (***N***=57908)	Post-intervention % (***N***=66623)	Type of change (positive +, negative −)	Magnitude of change %
Mandari	70.0	79.8	**+**	9.8
Tinga	70.4	82.7	**+**	12.3
Bole	83.7	80.5	**−**	(3.2)
Mankuma	68.0	86.0	**+**	18.0
Bamboi	98.9	99.0	**+**	0.1
Jama	96.9	98.8	**+**	1.9
Overall coverage district coverage	82.0	83.4	**+**	**1.4**
Self-reported adverse drug reaction^b^	**0.13**	**0.02**		

^a^This secondary data is from Bole District Health Administration neglected tropical diseases database

^b^This is per the number of people who ingested the drug

### Effectiveness

#### Knowledge of lymphatic filariasis

Most of the qualitative study respondents were able to describe lymphatic filariasis (LF) based on the signs and symptoms exhibited by patients. Others also relied on the causes of the disease in explaining what the disease is. The following are some responses to the question of what LF is:

…it is a disease that affects the human body and allows some fungi to grow on the affected part, and at a point, you will see the affected area growing to an abnormal position. Sometimes if it is the foot, you will see that foot is extraordinary fatter than the other foot, if it is the hand too, the same thing. But most people I have seen being affected by this disease have it on foot and hardly have I seen somebody being affected by the arm (Community leader, Mankuma).

It is a disease that when a mosquito bites a person who is not immunised and comes to bite you, then you also become infected (CDD, Mandari).

…it is a disease that affects the lymph system, which is mostly caused by mosquito. Mostly when it does happen, it is characterised by swelling, and the body parts it affects are the extremities and the scrotum and other parts of the body (Health worker, Tinga).

#### Knowledge on lymphatic filariasis

The survey result presented in Table 3 shows a significant difference in knowledge about LF among the study participants during the two phases of the study. The comprehensive knowledge of LF among the participants during the post-intervention phase of the study is higher compared to the pre-intervention phase.

**Table 3:** Knowledge about lymphatic filariasis before and after intervention among community members in Bole District

	Pre-intervention phase, ***N***=222		Post-intervention, ***N***=224		***P***-value
	Frequency	Percentage	Frequency	Percentage	
**Have correct knowledge of how LF is acquired**					<0.001
Yes	75	33.9	200	89.2	
No	147	66.1	24	10.8	
**Have correct knowledge of signs associated with LF**					<0.001
Yes	127	57.1	217	96.9	
No	95	42.9	7	3.2	
**Have correct knowledge of LF prevention**					<0.001
Yes	82	37.1	146	65.3	
No	140	63.0	78	34.7	
**Misconception about how LF is acquired**					<0.001
Yes	131	59.2	35	15.5	
No	91	40.8	189	84.5	
**Comprehensive knowledge of LF**					<0.001
Yes	70	31.5	178	79.7	
No	152	68.5	46	20.3	

#### Knowledge of lymphatic filariasis mass drug administration

Similar results were seen in the quantitative arm of the study, as shown in Table 4. There is a difference in the level of knowledge about the MDA between the study participants during the pre- and post-CEQI intervention phases. Participants in the post-intervention phase showed a high level of knowledge and participation in the MDA as compared to the pre-intervention phase. Adherence to the direct observation treatment (DOT) is significantly higher in the post-intervention phase than in the pre-intervention phase. Fear of adverse drug reaction as the reason for the refusal to swallow the medicine was higher in the pre-intervention phase than in the post-intervention phase.

**Table 4:** Knowledge of lymphatic filariasis mass drug administration in pre- and post-intervention among community members in Bole District

	Pre-intervention phase, ***N***=222		Post-intervention phase, ***N***=224		***P***-value
Variables	Frequency	Percentage	Frequency	Percentage	
**Awareness of LF MDA**					<0.001
Yes	102	46.0	173	77.2	
No	120	54.1	51	22.8	
**Source of MDA information**					<0.001^a^
Radio	2	2.3	2	1.3	
Health workers	6	5.4	58	33.5	
Posters	0	0.00	12	6.7	
Family members	7	6.7	2	0.9	
Church/mosque	18	18.0	23	13.4	
Community volunteers	37	36.5	39	22.3	
Gong-gong	14	13.5	35	20.5	
Neighbours/friends	18	17.6	2	1.3	
**Was there any public education before MDA**					<0.001^a^
Yes	40	39.6	154	89.0	
No	51	50.0	18	10.4	
Do not know	11	10.4	1	0.6	
**Have you ever taken the LF drug**					<0.001^a^
Yes	98	44.1	210	93.8	
No	82	36.9	10	4.5	
Cannot remember	42	18.9	4	1.8	
**How was the drug administered**					<0.001^a^
Direct observation treatment	140	63.1	221	98.7	
Given to beneficiary to take at his/her convenience	82	36.9	3	1.3	
Why some community ‘members’ refusal to ingest drug					<0.001^a^
Fear of side effects	143	64.4	140	62.5	
Level of knowledge of the disease	20	9.0	10	4.5	
Do not think they will get the disease	17	7.7	9	4.0	
Think only sick people should take the drugs	15	6.8	6	2.7	
Too many drugs	18	8.1	16	7.1	
Religious beliefs/superstition that oppose medication	6	2.7	3	1.3	
Taking other medication	2	0.9	19	8.5	
Have taken the drugs far too many times	1	0.5	21	9.4	

^a^Fisher’s exact test

#### Perception of the respondents on the cause of LF

Most respondents mentioned being bitten by a mosquito as a cause of the disease. However, two respondents (a drug distributor and community leader) said they had no idea about what causes the disease. There were speculations by one respondent that the disease is caused by witchcraft.

I don’t know what causes the disease, but some said it is caused by witches (Community leader, Jama).

Regarding preventive methods available for treating LF, most of the respondents indicated that taking the MDA drugs helps prevent the disease.

…health staffs have made us aware that if you take the drugs consistently, then you can prevent it (CDD, Jama).

…the only thing is to be taking the drugs always (CDD, Mandari).

…if you know you don’t have elephantiasis, and they are sharing the medicine, and you refuse to take them, it can’t prevent it unless you have taken the drug (Community leader, Bole).

The study participants were knowledgeable about the signs and symptoms of the disease. The commonest sign mentioned by respondents was swollen limbs or legs. Others were itchiness, rashes, headaches, frequent diarrhoea, dizziness, fever, fear of light, enlargement of the testes, reddish eyes, body pains and weakness in the body.

…you see one of the legs bigger than the other or very big testicles (CDD, Jama).

...sometimes I see the fellow having reddish eye and itchy body (Community leader, Mankuma).

#### Broad understanding of the MDA

According to respondents, the MDA is the distribution or administration of drugs to all community members (both LF patients and non-patients), to prevent and eradicate lymphatic filariasis. This is usually done using volunteers from the communities with supervision and monitoring from health workers.

We do the exercise because we don’t want anyone to get the disease in addition to those who already have it. It is an attempt to eradicate the disease (CDD, Bamboi).

...it has to do with we giving people drugs in our communities so that they are protected from getting the disease... mostly it happens once in a year... we go around to distribute the drugs... mostly we use the volunteers then the health workers do the monitoring and supervision (Health worker, Tinga).

Although all the respondents agreed that drugs given during the MDA are to prevent LF, some CDDs were also of the view that the drugs are capable of curing blindness and other diseases. It is worth noting that some community members were usually eager to ingest the drug because the drug enhances their sexual performance.

The medicine makes you active in whatever you are doing. Some community member said they could have sex with their wives and husbands very well (CDD, Mandari).

### Adoption and implementation

We learnt from the respondents that other activities undertaken before the 2017 MDA included informing the communities about the MDA via a town criers, sensitisation in churches, mosques, community information centres and sometimes on local radio stations. On a few occasions, durbars were held to educate community members on the LF disease and the importance of MDA. The endemic communities were also educated on reasons for the MDA and possible side effects of the drugs. Some respondents also reported that they used door-to-door approach to announce dates and to sensitise households. The reasons for the announcement by drug distributors were to ensure that people make themselves available on distribution days as most community members were farmers who sometimes spent nights on their farms.

…we pre-inform the people. This is because sometimes, some people go to spend the whole day and nights on the farm. Sometimes we even go to churches to make announcements with regards to the exercise so that the people are overly aware of the exercise (CDD, Jama).

It is important to note that most drug distributors disclosed that the focus was usually on announcements of dates rather than sensitisation. Very few of them mentioned sensitisation on the disease, possible side effects and how to handle such activities they undertook before the commencement of the MDA. Health workers who participated in the study, on the other hand, emphasised community mobilisation through durbars and meetings with stakeholders for sensitisation as one of the primary activities undertaken before the MDA began. This clearly shows that social mobilisation and sensitisation before and during the MDA is the health workers’ responsibility. There is an indication that community leaders were involved in the last MDA exercise, as shown in the quotes.

We do what we call social mobilisation, and we get the stakeholders to involve. We have a meeting with the stakeholders, and we discuss what we about to do, and we let them know the reason for carrying the exercise because without the stakeholders we can’t get the community to participate in the exercise... we have the volunteers involved in the meeting. We orientate them... we then send the information across to enforce the community sensitisation after the meeting (Health worker, Tinga).

The last time we were informed about the exercise, we beat the ‘gong-gong’ in the community to inform everyone about the exercise and when it will take place. We then advise them to make sure they avail themselves to take the medication. We also advise and encourage them to stay away from alcohol on the day of the exercise to avoid any complications (Community leader, Mankuma).

According to the participants, the drug distribution lasted between 1 and 2 weeks. Alcoholics, pregnant women, under-heights, lactating mothers and seriously sick people were excluded from the exercise. In cases where household members were unavailable to take the drugs or were unable to ingest the medicine for one reason or the other (sometimes because they were drunk), the house or structure was noted and revisited. The height of household members was measured to determine the dosage of medicine to be given.

...we have the measuring stick we use to check their height so when we establish that the person can take the drug, we make sure the person takes the drug in our presence...we don’t give it to you to take later (CDD, Tinga).

There is a strong indication that the Directly Observe Treatment (DOT) strategy was adhered to by the CDDs, as indicated in the following quotes:

...for some of them I fetch water, and they swallow the medicine there... especially those who didn’t take for the previous years… so they take it on the spot before I leave the house (CDD, Tinga).

...we make sure they take medicine in front of us so that they don’t go and throw the drugs away (CDD, Bole).

The participants revealed several differences, which made the most recent exercise more effective compared to previous ones. Some of the differences mentioned include detailed training for CDDs, increased incentive for CDDs, supportive supervision, assurance of free treatment of adverse drug effects by health service, a higher level of participation and community sensitisation.

The respondents indicated that many community members agreed to ingest the medication in the most recent (2017) MDA exercise compared to previous ones. They mentioned that they had to work hard in convincing community members to ingest the drugs in previous exercises, and it was a different case in the most recent MDA program. On some occasions, we were informed that community members went to the homes of the drug distributors or asked about the drugs even before the commencement of the MDA program. Some drug distributors also mentioned that they faced a minimal challenge in getting community members to comply on distribution day because they were eager and ready to participate in the MDA exercise.

Formerly they will say they don’t know you, but now they say you have not come to my house to give me the medicine (CDD, Mandari).

One major improvement is that we were given the assurance that if anyone faces any adverse effect after taking the drugs, they can report at the health facility, and they would be treated for free, and this boosted the confidence of the people in the drugs (Bole, Tinga).

Previously, people used to reject the drug and will never take it no matter what you say or do... but during the last distribution, people were in their houses waiting for me to bring the drug (CDD, Mankuma).

As I said earlier, the willingness of community members to ingest the medicine was very high in the last exercise...those who used to complain of drug reactions have now seen the benefits because we always convince them that the drug reactions they experience are because the organisms that cause the disease is already in their body, and so the drug is working on it, and so they need to continue taking the drugs to completely kill the organisms...previously, people used to reject the drug and will never take it no matter what you say or do (CDD Tinga).

A higher level of compliance in the new exercise, according to some CDDs, was due to the claims that the MDA drugs make one more physically active and enhance sexual performance. There was also an indication that community members understood the importance of MDA exercise and were willing to participate in the activity, which made work easy for CDDs. Pictorial evidence was shown to community members on the signs and symptoms of LF. It influenced compliance as it induced some fear and insight into the reality of the disease among community members who were likely to reject the drugs.

they say when you take it (the medicine), it makes you active in whatever you are doing and some also say they can sleep with their wives and husbands very well (CDD, Mandari).

The compliance was better now because they understood the exercise and so they were willing to swallow the drugs... sometimes those who were absent even trace me to come for theirs when they return (CDD, Bamboi).

During the previous drug distribution they were not willing to swallow, but last year they were willing due to the photos that we were holding (CDD, Tinga).

Another difference between the recent MDA and previous ones was the intense training volunteers received before the distribution exercise. Because of this training, respondents reported that they were able to engage community members better. In instances where refusals were imminent, they were able to convince community members to take the drugs.

...now what we do is that when the fellow wants to refuse to take the drugs, we explain the benefit of the drugs to them. But formerly, if the person doesn’t want to take the drug, we leave (CDD, Mandari).

...but now because of the intensive training, we take our time to explain to them, so most of them agreed (CDD, Tinga).

Even though respondents said they used different channels (religious centres, gong-gong beating, door-to-door approach) to sensitise community members and announce distribution dates, introducing some new channels such as the use of community information centres and radios were new additions. These new additions reached a wider population compared with the traditional channel of the house-to-house announcement and gong-gong beating. A CDD had this to say:

We used to go from house to house to inform people, but now there are information centres around that we were encouraged to use... you can just go there and make the announcements and people hear it from their homes (CDD, Jama).

Unlike in previous exercises where drugs were just administered without measures for treating adverse side effects, respondents felt that provisions made in the recent exercise for free treatment of adverse reactions helped. Community members were informed on where to seek free health care should they have any adverse drug reaction And that motivated community members to ingest the drugs. As shown in the following quote:

One other improvement is that we were given the assurance that if anyone faces any adverse effect after taking the drugs, they can report at the health facility, and they would be treated for free, and that boosted the confidence of the people in the drugs (CDD, Jama).

Respondents also mentioned an increase in allowances for volunteers and supervision in the most recent exercise compared to previous MDA exercises. These motivated drug distributors to give their best.

...well, the money we were given in the last MDA exercise was an increment from the previous ones (CDD, Bamboi).

In fact, before God and man, there was a slight increment in the allowances that are given to the volunteers in this last MDA exercise, and this encouraged us to work harder (CDD, Tinga).

During the last exercise, the MDA officers were around to supervise us, so when you are going astray, they will correct you this helped us a lot (CDD, Mandari).

Some distributors revealed that coverage in some communities in the recent MDA program was affected by community members’ migration to other parts of the country and so were not accessible. The results also show that the timing of the MDA being Islamic fasting period also contributed to the low participation in some communities.

I realised that the number of people covered in the previous drug distribution was more than in the recent exercise because many of the people who had written their names in some of the houses were nowhere to be found because most of them had either moved out or travelled (CDD, Bole).

...you know Bole is a Muslim community, and the drug was distributed at the time of Ramadan so the people fasting could not take part (Health Worker, Bole).

The fight against ‘galamsey’ (illegal mining of gold) caused a lot of out-migration in some communities, hence resulting in several community members not available.

For my community, the coverage was low because previously, there were a lot of galamsey workers around and so the number of people was very high. But now that the galamsey has been stopped, all those people have moved away, and so it has caused a decrease in the number of people (CDD, Bole).

One challenge is that, since this is a galamsey area, you go and register a lot of people, but during the drug distribution they tell you they have moved out (Health worker, Bole).

There were reports by CDDs that they could not complete or cover the areas assigned to them within the stipulated time of the MDA. This was partly because of the increase in compliance or acceptance, which meant more people to attend. Some of the areas within the district had seen some development and population increase. Thus, some households were left out of the exercise.

Other community members rejected MDA drugs due to misconception, fear of side effects and some health facilities’ unwillingness to treat such cases for free. The nearest health facility’s distance to report adverse drug reaction was also assigned as a reason for the drug’s refusal. CDDs outlined these as challenges encountered during the last MDA exercise.

Because a lot of people including some visitors were ready to take the drug, I spent more time attending to people in some houses, and I was not able to visit all the houses in my area. (CDD, Mankuma).

…some of the community people said when they ingest the drug, their legs become swollen and I tell them if it has swollen they should come to the health centre, and they will give them drugs and they said when they go to the health facility, the nurses will not attend to them (CDD, Tinga).

Some community members refused the drugs because they did not trust the volunteers, accusing them of accepting bribes to administer deadly drugs.

There were even people who would refuse to take the drugs and then ask you to leave their house... they say you have been given money to come and give them drugs for which they may even die after taking (CDD, Mankuma)*.*

Lack of logistics such as transportation and protective boots to enable drug distributor access remote areas were among the challenges encountered.

One thing they can do to help us is provided wellington boots and raincoats for us to be able to reach the communities that have muddy access and when it rains on the way (CDD, Bamboi).

Community members also had unrealistic demands and demanded incentives such as mosquito nets that were not part of the program.

Everywhere we go they talk about mosquito nets. They said we always talk about LF and oncho, but we don’t give them mosquito nets so they will not take the drugs (CDD, Tinga).

### Maintenance

Due to the short duration of the CEQI intervention, detailed evaluation of maintenance was difficult to assess and determine its sustainability. However, we reviewed barriers and possible solutions to enable successful implementation and sustainability of the intervention from the study participants’ perspective.

Because of the challenges encountered during the last MDA exercise, respondents suggested that the MDA exercise should be conducted during the dry season when there will be no farming activities to ensure accessibility to communities and individual household members’ availability. The interval between MDA exercises was reported to be too long, and some CDDs suggested that the interval should be shortened.

My suggestion is the interval between the MDAs is too long because people continue asking when is the tablet coming (CDD, Jama).

To ensure the sustainability of the intervention, the CDDs suggested that more knowledgeable people (health worker) should accompany them to authenticate their credibility during the drug distribution. According to some CDDs, community members knowing volunteers are not medical personnel hence disregard information they give and consider it as inaccurate and incredible.

We need more help to encourage us to do the job because if it only we the volunteers when we are talking to them, they won’t listen to us. They will say you have never been to school, and you are coming to give medicine to us (CDD, Bole).

CDDs also recommended better remuneration, provision of incentives and motivation for community members.

I think providing us with bicycles and increasing the allowance will help us reach the very far communities easily... and distributing bed-nets to the community members will also help (CDD, Bamboi).

One major threat to the sustainability of the CEQI intervention observed is the frequent transfer of health staff within the district. Between 2017 to December 2018, key health worker (senior disease control officers and district director) has been transferred. These health workers were vital members of the CEQI implementation team and management members of the district health administration.

## Discussion

The study aimed to assess the effect of implementing CEQI intervention on the implementation of LF MDA in the Bole District of Northern Ghana using RE-AIM. The RE-AIM framework provided a structure to evaluate the impact of the CEQI towards the elimination of the LF as a public health problem in hotspot districts in Ghana.

The evaluation findings showed improved social mobilisation and sensitisation, knowledge about LF and MDA process, willingness to ingest the medication, and adherence to the DOT Strategy. We observed a 6.3% increase in the district MDA coverage and 0.03% reduction in self-reported adverse drug reaction.

However, the interventions did not have the desired effects on MDA coverage in Bole sub-district. This was due to the timing of the MDA and peri-urban nature of the sub-district. Bole sub-district is a Muslim-dominated population; administering drugs to the people around the Muslim fasting period made it difficult for most people to ingest the medicine, causing the decline in the MDA coverage. Secondly, social mobilisation and sensitisation strategy, which works well in rural areas, did not work in Bole sub-district due to the semi-urban nature of the sub-district.

These observations reveal the contextual complexities of working in semi-urban/urban communities (as demonstrated in Bole sub-district). Health workers at the local level understand their system and difficulties much better than the national level team.

The evaluation revealed a substantial improvement in knowledge, risk perceptions and understanding of LF and the MDA. Comparable observations have been made elsewhere [4, 29, 30]. They reflect the need to create awareness through social mobilisation and sensitisation using evidence-based context-specific strategies and involvement of community leaders. Other key stakeholders in deciding on the strategies and methods to improve MDA activities towards LF control based on initial needs assessment [5, 11, 31].

Intensive training and motivation for CDDs were also a particularly important factor determining the success of the ongoing LF MDA in the study district, as shown elsewhere [4, 32].

The nature of contextual complexities and dynamics of populations in LF endemic areas may require that MDA activities be designed considering contextual factors based on WHO-recommended strategies [4, 5, 33]. Social mobilisation and sensitisation materials and strategy should focus on the local context, need-based, social and culture structures [4, 34]. This study also revealed that the involvement of community leaders, intensive training of CDDs and employing context-specific evidence-based social mobilisation and sensitisation strategies led to an improvement in the knowledge, attitude and practices relating to the disease, reduction in misconception regarding LF and MDA activities and willingness to ingest the LF medicine in the study area.

The fear of adverse drug reaction associated with the LF medication was an important factor influencing the decision to take the MDA drugs and has been identified in several studies elsewhere [9, 35] and the study area [5]. In this study, the fear of adverse reactions improved after the implementation of QI intervention, indicating that the challenge of fear of adverse drug reaction in control of LF can be eradicated through the implementation of context-specific educational strategies, building confidence in the populace, the outlining of the reasons for the occurrence of drug reactions, and what to do when they occur as indicated in this study. While misconception about the drug (enhances sexual performance) still exists in some areas, continuous appropriate education and advocacy may help overcome this challenge to avoid drug abuse in some areas. Continuous appropriate education and advocacy may help overcome this challenge to avoid drug abuse.

This study has reinforced the importance of applying the RE-AIM framework to evaluate public health intervention using a mixed methods approach.

The increasing advocate for public health studies to apply qualitative and mixed methods in health services delivery research has been demonstrated in other studies [36–40]. Guidance is scarce in the literature on applying qualitative methods in the RE-AIM framework [41]. Therefore, the inclusion of qualitative approaches in this study is vital to the full application of the RE-AIM model in assessing public health interventions [15, 22]. The combination of qualitative and quantitative methods helped to evaluate the CEQI intervention when very complex and unbiased quantitative data are not available or feasible [41].

Although the qualitative methods may not represent the entire study population, it added depth and meaning to facilitate understanding [41]. This study’s methods have also enriched the understanding and conclusion of the CEQI intervention evaluation. This approach can guard against the wrong assumption that an intervention or method did not work when implementing failure [42, 43]. Finally, the approaches used in this study have offered insight into how to guard against implementation failures in the future application of the CEQI intervention. Mixed methods can both enhance and advance the effective application of the RE-AIM [41].

### Limitations

This study has some limitations. Social desirability bias could be a limitation to the study. Some study participants might have withheld what they thought to be harmful practices during the implementation of the CEQI from the researchers. The duration of the intervention (one round of implementation) does not provide knowledge of the long-term effect and difficulties within the setting and with participants to be thoroughly studied.

Due to the pilot nature of the CEQI intervention, detailed evaluation of maintenance was difficult to assess and determine the intervention’s sustainability. We were unable to thoroughly verify the quality of the MDA coverage data used in this study.

The CEQI intervention has been implemented for only one round of MDA. The intervention was limited to only one district in Northern Ghana, hence limits the generalisability of the findings.

## Conclusion

The findings of this study at the district and sub-district level, coupled with reach, effectiveness and adoption at the participant level, suggest that the CEQI intervention is feasible to implement in LF hotspot districts based on initial context-specific needs assessment.

Although there is an improvement in coverage, compliance, knowledge about the disease and MDA activities in the study area, the study further emphasised the need to improved context-specific social mobilisation and sensitisation focusing on the safety of medicines and the importance of MDA before and during the drug distribution exercises.

## Data Availability

The dataset supporting the conclusions of this article is included in the article.

## Abbreviations

CEQI: Context-specific evidence-based quality improvement
CDD: Community drug distributors
DOT: Direct observation treatment
GSELF: Global strategy to eliminate lymphatic filariasis
GFEP: Ghana Filariasis Elimination Program
IDI: In-depth interviews
LF: Lymphatic filariasis
MDA: Mass drug administration
NTD: Neglected tropical diseases
RE-AIM: Reach, Effectiveness, Adoption, Implementation, and Maintenance

## References

[1] Zeldenryk LM, Gray M, Speare R, Gordon S, Melrose DW (2011). The emerging story of disability associated with lymphatic filariasis: a critical review. PLoS Negl Trop Dis.

[2] Molyneux D (2003). Lymphatic filariasis (elephantiasis) elimination: a public health success and development opportunity. Filaria J.

[3] WHO: Guideline – alternative mass drug administration regimens to eliminate lymphatic filariasis. 2017d. 29565523

[4] Biritwum N-K, Garshong B, Alomatu B, de Souza DK, Gyapong M, Kyelem D (2017). Improving drug delivery strategies for lymphatic filariasis elimination in urban areas in Ghana. PLoS Negl Trop Dis.

[5] Manyeh AK, Ibisomi L, Baiden F, Chirwa T, Ramaswamy R (2019). Using intervention mapping to design and implement quality improvement strategies towards elimination of lymphatic filariasis in Northern Ghana. PLoS Negl Trop Dis.

[6] Biritwum N-K, de Souza DK, Marfo B, Odoom S, Alomatu B, Asiedu O (2017). Fifteen years of programme implementation for the elimination of lymphatic filariasis in Ghana: Impact of MDA on immunoparasitological indicators. PLoS Negl Trop Dis.

[7] Babu BV, Mishra S (2008). Mass drug administration under the programme to eliminate lymphatic filariasis in Orissa, India: a mixed-methods study to identify factors associated with compliance and non-compliance. Trans Roy Soc Trop Med Hyg.

[8] Cantey PT, Rao G, Rout J, Fox LM (2010). Predictors of compliance with a mass drug administration programme for lymphatic filariasis in Orissa State, India 2008. Trop Med Int Health.

[9] Krentel A, Fischer PU, Weil GJ (2013). A review of factors that influence individual compliance with mass drug administration for elimination of lymphatic filariasis. PLoS Negl Trop Dis.

[10] Lemoine JF, Desormeaux AM, Monestime F, Fayette CR, Desir L, Direny AN, Baker M (2016). Controlling neglected tropical diseases (NTDs) in Haiti: implementation strategies and evidence of their success. PLoS Negl Trop Dis.

[11] Njomo D, Amuyunzu-Nyamongo M, Mukoko D, Magambo J, Njenga S (2012). Social mobilization and compliance with mass treatment for lymphatic filariasis elimination in Kenya. Afr J Health Sci.

[12] Glasgow RE, McKay HG, Piette JD, Reynolds KD (2001). The RE-AIM framework for evaluating interventions: what can it tell us about approaches to chronic illness management?. Patient Educ Couns.

[13] Finch CF, Gabbe BJ, Lloyd DG, Cook J, Young W, Nicholson M, Seward H, Donaldson A, Doyle TLA (2011). Towards a national sports safety strategy: addressing facilitators and barriers towards safety guideline uptake. Inj Prev.

[14] Dzewaltowski DA, Estabrooks PA, Glasgow RE (2004). The future of physical activity behavior change research: what is needed to improve translation of research into health promotion practice?. Exerc Sport Sci Rev.

[15] Kessler RS, Purcell EP, Glasgow RE, Klesges LM, Benkeser RM, Peek CJ (2013). What does it man to “employ” the RE-AIM model?. Eval Health Prof.

[16] Akers JD, Estabrooks PA, Davy BM (2010). Translational research: bridging the gap between long-term weight loss maintenance research and practice. J Am Diet Assoc.

[17] Kahwati LC, Lance TX, Jones KR, Kinsinger LS (2011). RE-AIM evaluation of the veterans health Administration’s MOVE! weight management program. Transl Behav Med.

[18] Dunton GF, Lagloire R, Robertson T (2009). Using the RE-AIM framework to evaluate the statewide dissemination of a school-based physical activity and nutrition curriculum: “Exercise Your Options”. Am J Health Promot.

[19] Finch C (2012). Implementing and evaluating interventions. In injury research: theories, methods, and approaches.

[20] Li F, Harmer P, Glasgow R, Mack KA, Sleet D, Fisher KJ, Kohn MA, Millet LM, Mead J, Xu J (2008). Translation of an effective Tai Chi intervention into a community-based falls-prevention program. Am J Public Health.

[21] Estabrooks PA, Bradshaw M, Dzewaltowski DA, Smith-Ray RL (2008). Determining the impact of Walk Kansas: applying a team-building approach to community physical activity promotion. Ann Behav Med.

[22] Gaglio B, Shoup J, Glasgow RE (2013). The RE-AIM framework: a systematic review of use over time. Am J Public Health.

[23] Payne JM, France KE, Henley N, D’Antoine HA, Bartu AE, O’Leary CM, Elliott EJ, Bower C, Geelhoed E (2011). RE-AIM evaluation of the alcohol and pregnancy project: educational resources to inform health professionals about prenatal alcohol exposure and fetal alcohol spectrum disorder. Eval Health Prof.

[24] Vick L, Duffy SA, Ewing LA, Rugen K, Zak C (2013). Implementation of an inpatient smoking cessation programme in a veterans affairs facility. J Clin Nurs.

[25] Oliver M (2000). An introduction to the evaluation of learning technology. Edu Technol Soc.

[26] Lwanga SK, Lemeshow S (1991). Sample size determination in health studies’ a practical manual. World Health Organisation.

[27] Offei M, Anto F (2014). Compliance to mass drug administration programme for lymphatic filariasis elimination by community members and volunteers in the Ahanta West District of Ghana. J Bacteriol Parasitol.

[28] Manyeh AK, Ibisomi L, Ramaswamy R, Baiden F, Chirwa T (2020). Exploring factors affecting quality implementation of lymphatic filariasis mass drug administration in Bole and Central Gonja Districts in Northern Ghana. PLoS Negl Trop Dis.

[29] Nandha B, Sadanandane C, Jambulingam P, Das P (2007). Delivery strategy of mass annual single dose DEC administration to eliminate lymphatic filariasis in the urban areas of Pondicherry, South India: 5 years of experience. Filaria J.

[30] Yirga D, Deribe K, Woldemicheal K, Wendafrash M, Kassahun W (2010). Factors associated with compliance with community directed treatment with ivermectin for onchocerciasis control in Southwestern Ethiopia. Parasites Vectors.

[31] Mwakitalu ME, Malecela MN, Pedersen EM, Mosha FW, Simonsen PE (2013). Urban lymphatic filariasis in the city of Tanga, Tanzania, after seven rounds of mass drug administration. Acta Tropica.

[32] Njomo DW, Amuyunzu-Nyamongo M, Magambo JK, Ngure PK, Njenga SM (2012). Factors associated with the motivation of community drug distributors in the Lymphatic Filariasis Elimination Programme in Kenya. South Afr J Epidemiol Infect.

[33] Njomo DW, Mukoko DA, Nyamongo NK, Karanja J (2014). Increasing coverage in mass drug administration for lymphatic filariasis elimination in an urban setting: a study of Malindi Town, Kenya. PLoS One.

[34] Molyneux DH, Hopkins A, Bradley MH, Kelly-Hope LA (2014). Multidimensional complexities of filariasis control in an era of large-scale mass drug administration programmes: a can of worms. Parasites Vectors.

[35] Cantey PT, Rout J, Rao G, Williamson J, Fox LM (2010). Increasing compliance with mass drug administration programs for lymphatic filariasis in India through education and lymphedema management programs. PLoS Negl Trop Dis.

[36] Dowding D. Best practices for mixed methods research in the health sciences John W. Creswell, Ann Carroll Klassen, Vicki L. Plano Clark, Katherine Clegg Smith for the Office of Behavioral and Social Sciences Research; Qualitative Methods Overview Jo Moriarty. Qualitative Social Work. 2013;12(4):541–5. 10.1177/1473325013493540a.

[37] Curry LA, Krumholz HM, O’Cathain A (2013). Mixed methods in biomedical and health services research. Circ Cardiovasc Qual Outcomes.

[38] Plano Clark VL (2010). The adoption and practice of mixed methods: U.S. trends in federally funded health-related research. Qual Inq.

[39] Miller WL, Crabtree BF, Harrison MI (2013). Integrating mixed methods in health services and delivery system research. Health Serv Res.

[40] Palinkas LA, Cooper (2017). BR: Mixed methods evaluation in dissemination and implementation science.

[41] Holtrop JS, Rabin AB, Glasgow RE (2018). Qualitative approaches to use of the REAIM framework: rationale and methods. BMC Health Serv Res.

[42] Bellg AJ, Borrelli B, Resnick B (2004). Enhancing treatment fidelity in health behavior change studies: best practices and recommendations from the NIH behavior change consortium. Health Psychol.

[43] Barrera M, Castro FG, Strycker LA (2013). Cultural adaptations of behavioral health interventions: a progress report. J Consult Clin Psychol.

